# Nutritional support for severe malnutrition in newly diagnosed Crohn’s disease: a case report

**DOI:** 10.3389/fnut.2026.1922931

**Published:** 2026-09-09

**Authors:** Daniele Canova, Linda Cingolani, Gessica Schiavo

**Affiliations:** 1 Department of Gastroenterology, San Bortolo Hospital, Vicenza, Italy; 2 Department of Clinical Nutrition, San Bortolo Hospital, Vicenza, Italy

**Keywords:** biological therapy, Crohn’s disease, enteral nutrition, malnutrition, parenteral nutrition

## Abstract

Crohn’s disease (CD) and ulcerative colitis are chronic inflammatory bowel diseases (IBDs) characterized by relapsing exacerbations related to a dysregulated immune response, resulting primarily in gastrointestinal symptoms and also frequently in signs of chronic systemic inflammation. Malnutrition is a possible complication of IBD with a prevalence ranging from 20 to 85%, varying with disease activity, location, and age, and is recognized as having a multifactorial pathogenesis. Malnutrition in patients with IBD should be diagnosed early and treated appropriately because it can heavily affect patient prognosis, including complications, hospitalization rates, mortality, and overall quality of life. Therefore, patients with IBD should be screened for malnutrition at diagnosis and regularly thereafter. Moreover, assessing micronutrient deficiencies in patients with IBD is recommended ideally during clinical and biochemical remission. We reported the case of a 56-year-old female patient admitted to our department who reported chronic diarrhea for several months, along with recurrent abdominal pain, low-grade evening fever, nausea, hyporexia, and significant involuntary weight loss, with probable sarcopenia. Iron-deficiency anemia, an elevated platelet count, elevated inflammatory markers, and some oligoelement deficiencies were observed on blood tests. A diagnosis of extensive ileo-colonic CD (A3, L3, B1 according to the Montreal classification) complicated by a perianal abscess and simple intrasphincteric fistula, requiring surgery, was made during the hospitalization. As agreed with our nutritional team, given the severe malnutrition observed in patients at risk of refeeding syndrome, the patient received thiamine supplementation, minimal enteral feeding with oral nutritional supplements (ONS), and simultaneous parenteral nutrition with a ternary emulsion supplemented with vitamins and trace elements. Parenteral nutritional supplementation and ONS were continued for 2 months after discharge to improve the patient’s nutritional status. Subsequently, the patient was able to eat only natural foods, as advised by our hospital’s nutrition team. Finally, we briefly reviewed recommendations on malnutrition screening and enteral or parenteral nutrition in patients with IBD to reduce morbidity and mortality due to malnutrition.

## 1 Introduction

Crohn’s disease (CD) and ulcerative colitis (UC) are chronic inflammatory bowel diseases (IBDs) characterized by relapsing exacerbations related to a dysregulated immune response. Both genetic susceptibility and environmental factors, including diet, play essential roles in its pathogenesis. Dietary components can influence gut microbiota composition, immune regulation, and intestinal barrier integrity, thereby modulating disease onset, severity, and relapse (1).

There is evidence that specific dietary patterns can impact IBD outcomes (2–4); the prevalence of malnutrition in patients with IBD ranges from 20 to 85%, varying with disease activity, location, and age (5). A multifactorial origin can be recognized in the pathogenesis of malnutrition in IBD (5–7). The consequences of malnutrition in IBD may vary from weight loss with sarcopenia to impaired immune function, delayed wound healing, increased infection risk, and compromised response to therapy. Patients with IBD should be screened for malnutrition at diagnosis and regularly thereafter, as it is often related to greater disease activity and higher rates of hospitalization; therefore, once documented, it should be treated appropriately (7–10).

The ECCO Consensus recommends combining body mass index (BMI) with a measure of body composition, muscle function, or both. Techniques such as dual-energy x-ray absorptiometry (DEXA), computed tomography (CT), or magnetic resonance imaging (MRI) quantify muscle mass, while practical tools such as handgrip strength offer accessible ways to assess muscle function over time (11, 12). Moreover, assessing micronutrient deficiencies in patients with IBD is recommended ideally during clinical and biochemical remission (12).

## 2 Case description

A 56-year-old female patient reporting chronic diarrhea with five to seven bowel movements daily for 6 months, along with recurrent abdominal pain and low-grade evening fever, nausea, hyporexia, and significant involuntary weight loss during the previous 3 months (13 kilograms, corresponding to 23% of her previous weight), was referred to our Gastroenterology Unit. She also reported anal pain for 1 week.

As relevant medical history: in 2021, following the diagnosis of left mammary ductal cancer, the patient underwent neoadjuvant chemotherapy, mastectomy, and radiotherapy, and in 2022 she was diagnosed with Lynch syndrome through the detection of a mutation in the MSH2 gene.

At the time of admission to our Department, physical examination revealed severe underweight (BMI 14.8 kg/m^2^, with a weight of 42 kg and height of 167 cm) and a perianal painful tumefaction, whereas urgent blood tests showed normocytic anemia (hemoglobin, 80 g/L), iron deficiency (17 ug/dl), elevated platelet count (546 ×10^9^/L) and increased inflammatory markers (erythrocyte sedimentation rate, 104 mm/h; C-reactive protein [CRP], 74.3 mg/L; ferritin, 609 ug/L), a high fecal calprotectin level (9,022 ug/g), and normal calcium levels; serum albumin was reduced (3.1 g/dL).

A Nutritional Risk Score (NRS-2002) (13) of four and a Malnutrition Universal Screening Tool (MUST) score (14) of six were calculated, indicating a risk of malnutrition and therefore requiring nutritional management. The handgrip strength test (HST) showed a value of 14 kg (average of three measurements), while a calf circumference of 21.5 cm (average of three measurements) was measured, both suggestive of low muscle strength. The HST was implemented with a Hydraulic Hand Dynamometer, with the patient seated on a chair with armrests. The cut-off values employed are those recommended by the European consensus EWGSOP2 (European Working Group on Sarcopenia in Older People) to define low handgrip strength. Calf circumference was measured with a flexible tape along the widest part of the leg, while the patient was seated with leg relaxed and knee bent at a 90-degree angle. Our nutrition team diagnosed severe malnutrition using the Global Leadership Initiative on Malnutrition (GLIM) criteria (15).

Given the severe malnutrition, low BMI and baseline hypophosphataemia and hypokalaemia, the patient was classified as at high risk of refeeding syndrome according to ESPEN 2023 guideline (10). The initial caloric intake was 200 kcal/day and was progressively increased. Two months after discharge the patient introduced 2,580 kcal/day; phosphate, potassium, magnesium, glucose and fluid balance were monitored. No clinical signs of refeeding syndrome were observed during hospitalization.

**Figure 1:**
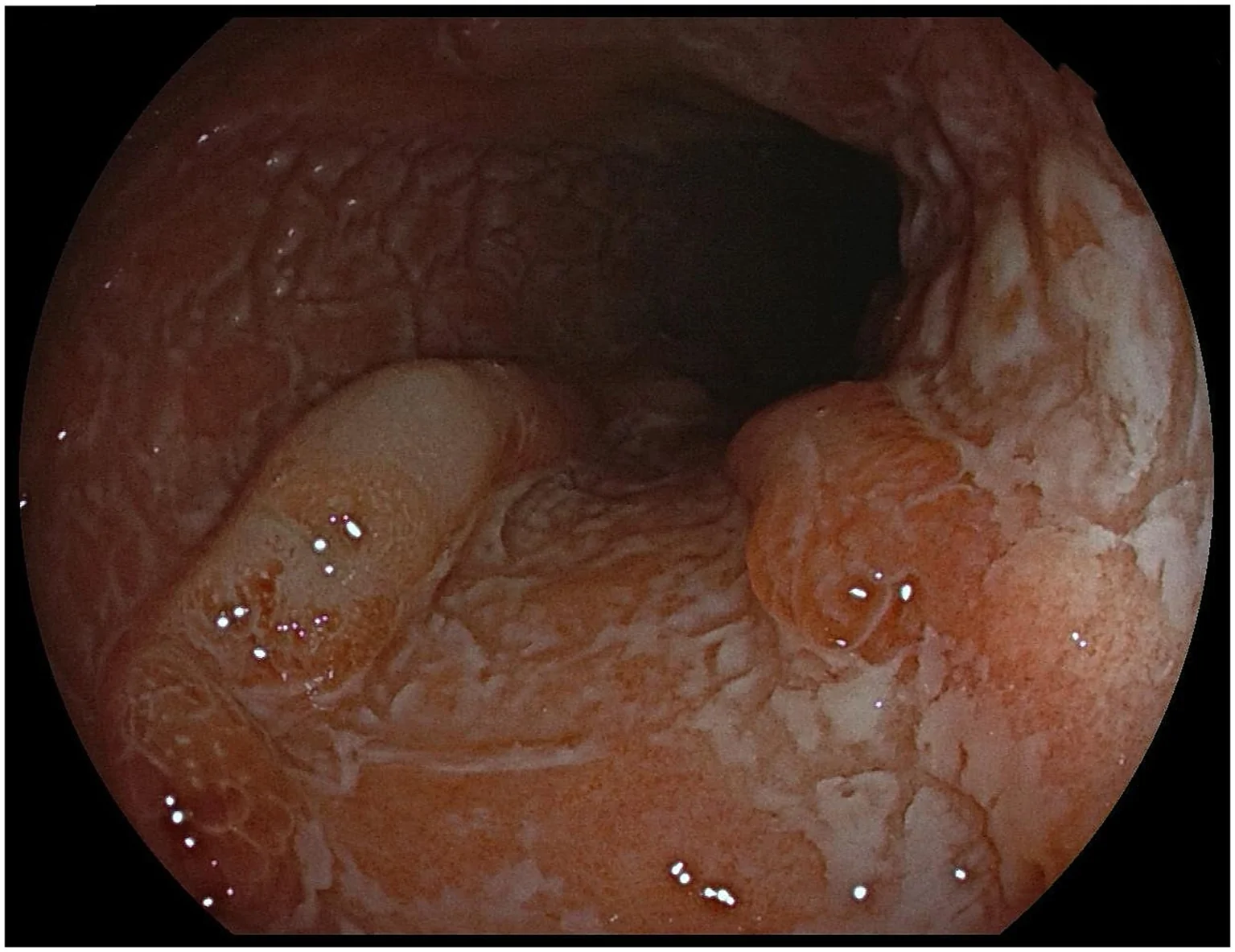
Colonoscopic view (sigmoid colon) consistent with severe colitis.

**Figure 2:**
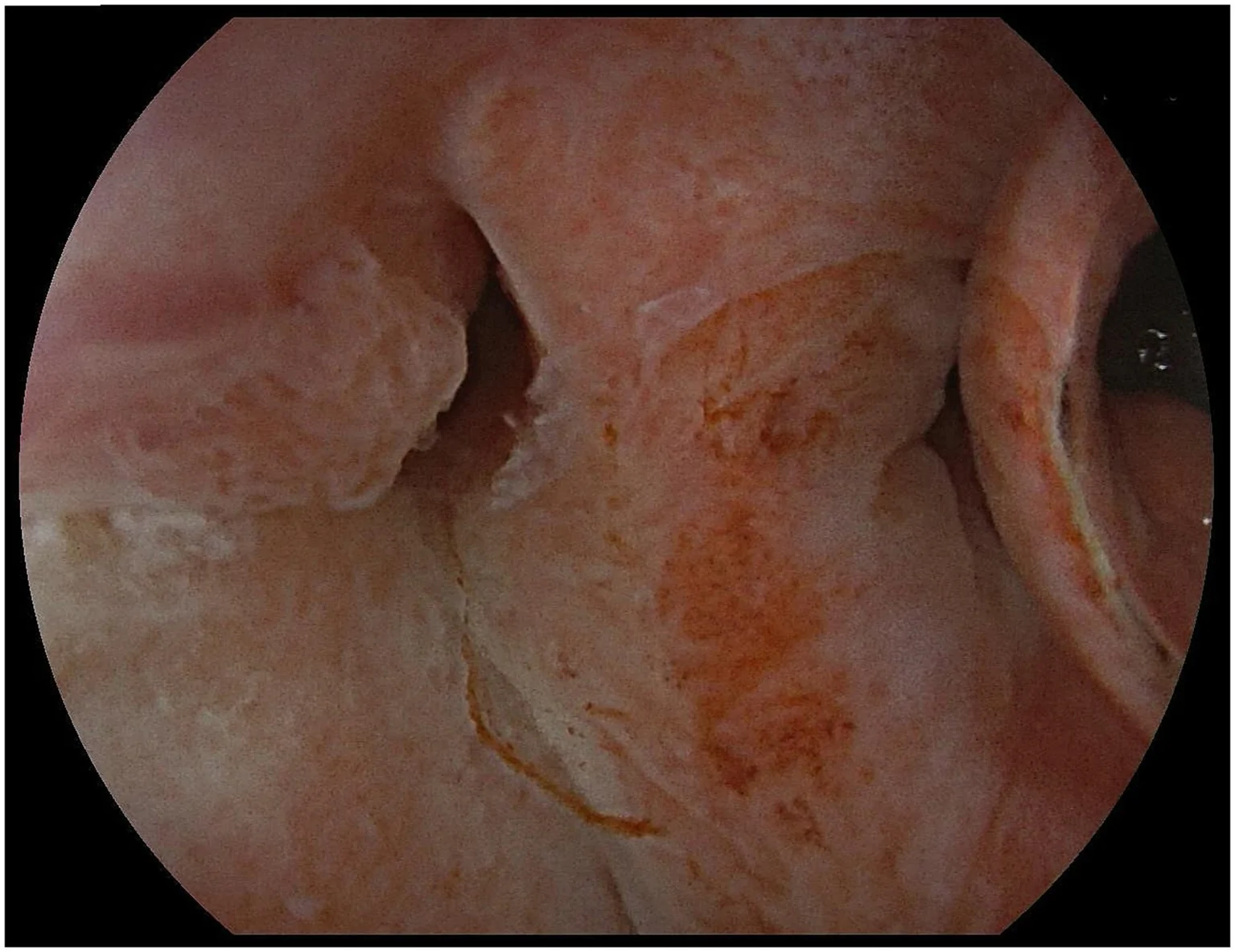
Colonoscopic view (rectal fistula).

During hospitalization, the patient underwent multiple assessments, including:

- Baseline nutritional status evaluation: total protein, 6.5 g/dL; albumin, 3.1 g/dL; prealbumin, 0.05 g/L; transferrin, 113 mg/dL; total cholesterol, 106 mg/dL; triglyceride, 107 mg/dL; phosphate, 0.6 mg/dL; potassium, 2.3 mmol/L; magnesium, 2.2 mg/dL; folic acid, 7 nmol/L; B12 vitamin, 46 pmol/L; zinc, 5.4 umol/L; and vitamin D, 28.7 nmol/L.
- Abdominal CT scan, showing colonic wall thickening, afferent vessel hyperemia, and inflammatory mesenteric lymphadenopathy.
- Esophagogastroduodenoscopy, showing antral gastropathy. Extensive duodenal and gastric biopsy mapping revealed no histological alterations.
- Colonoscopy, showing extensive colitis (Figure 1), distal ileitis (Simplified Endoscopic Activity Score for Crohn’s Disease [SES-CD] score: 23) and suspicion of a rectal fistula (Figure 2). Extensive biopsy mapping confirmed an ileo-colic inflammatory histological pattern consistent with CD.
- Magnetic resonance (MR) enterography and lower abdominal MRI documented diffuse large bowel wall thickening and an abscessed simple intrasfinteric perianal fistula.

**Figure 3:**
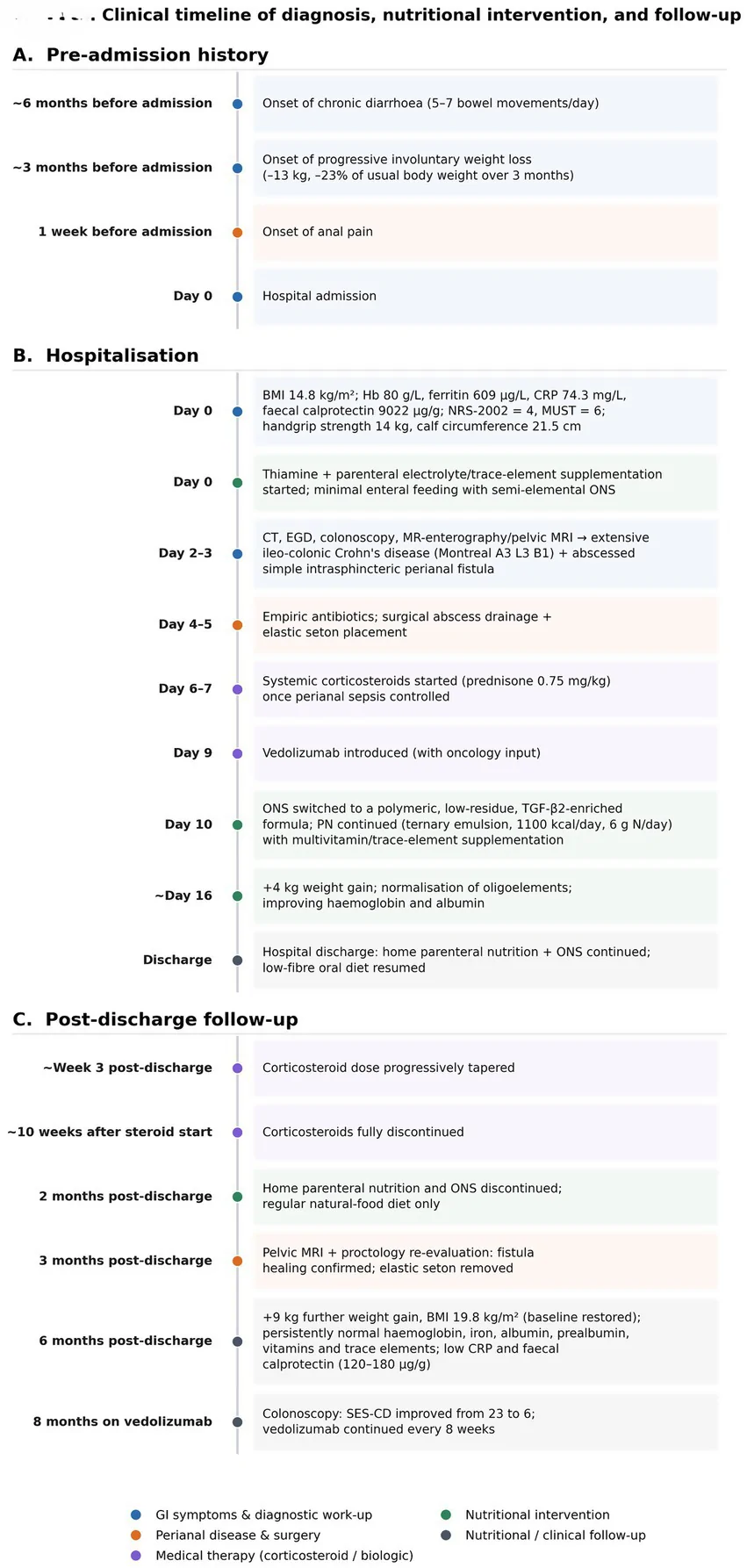
Clinical timeline of diagnosis, nutritional intervention, and follow-up. Chronological summary organized into **(A)** pre-admission history, **(B)** hospitalization, and **(C)** post-discharge follow-up, color-coded by clinical domain (GI symptoms/diagnostic work-up, perianal disease/surgery, medical therapy, nutritional intervention, and follow-up).

Therefore, empiric antibiotic therapy was initiated, while the patient underwent surgical examination, abscess drainage, and application of an elastic seton. Once the perianal disease was controlled, systemic steroid therapy was initiated (prednisone, 0.75 mg/kg) with subsequent rapid clinical improvement, and shortly after, in accordance with oncologists, a biotechnologic agent (Vedolizumab) was introduced (Figure 3).

Since her hospital admission, the patient has received intramuscular thiamine administration (100 mg each day within the first 3 days, followed by 300 mg via oral route daily in the next 3 months). Targeted micronutrient repletion was undertaken alongside nutritional support (detailed values of deficiencies are reported below): intravenous iron (1,000 mg) was administered first, followed by oral iron supplementation (30 mg daily for 3 months). Oral folate (5 mg/day for 3 months) and an initial intramuscular vitamin B12 injection (1,000 mg/day for 5 consecutive days) followed by sublingual maintenance (5,000 mcg/week for 3 months) were started. Oral vitamin D (100.000 UI every 4 weeks) was administered. Magnesium sulfate 1 g/10 mL every day for 5 days, oral monobasic phosphate 500 mg/day for 7 days and dibasic potassium 250 mg every day for ten days during hospitalization and for 3 weeks after discharge were given. All the oligoelements were administered separately from parenteral nutrition formulation.

The patient experienced persistent nausea and difficulty with oral intake. We used the HB equation with 1.2 stress factor and protein requirement: 1.2–1.5 g/kg/day. In accordance with our nutrition team, we started a minimal enteral feeding regimen with ONS using a normocaloric semielementary formulation (Peptamen 200 mL/day, energy 200 kcal, proteins 8 g). After 10 days, we changed to a nutritionally complete ONS, low-residue polymeric formula enriched with TGF-β2 (Modulen IBD powder: 100 g daily; 100 kcal/day, 3.6 g proteins). The ONS was supplemented with parenteral nutrition using a ternary emulsion, deliberately kept at a moderate caloric target as part of refeeding-syndrome prevention (corresponding to 1,100 kcal/day, 6 g N/day), with additional multivitamin, until discharge.

Over the subsequent days, we observed a progressive increase in oral intake, weight gain (4 kilograms), and normalization of serum levels of the main oligonutrients, with improvement in both hemoglobin and albumin levels. The patient gradually resumed regular oral intake on a low-fiber diet and with an enteral formulation. The daily steroid dosage was progressively decreased until it was discontinued after 10 weeks. Parenteral nutrition was progressively reduced and continued at home after hospital discharge. At discharge, home parenteral nutrition was prescribed (1,100 kcal/day, 6 N/day) for 3 days a week and daily ONS (Modulen IBD 100 g). We do not observe adverse effects.

Formal rescoring with the NRS-2002, MUST, or GLIM criteria was not repeated at hospital discharge; the patient’s clinical and biochemical trajectory (weight gain, improving hemoglobin and albumin) was consistent with improving, though not yet fully resolved, malnutrition at that time. Since discharge, weight has gradually increased: 4 kg in the first month and 2 kg in the second, with the usual weight being reached after 6 months (+10 kg).

## 3 Discussion

The prevalence of malnutrition in patients with IBD is difficult to estimate, ranging from 6 to 70% according to different definitions (16–18). It is generally associated with persistent disease activity, but it can also be observed in patients in clinical remission (10).

Patients with IBD and malnutrition are at increased risk of exacerbation of the IBD itself, increased morbidity and mortality (6, 10): therefore, patients with IBD need screening for malnutrition, followed by assessment and management. First, nutritional care includes the prevention of malnutrition and micronutrient deficiencies, as well as the treatment of malnutrition, to avoid complications and nutrition-related disorders (10).

Additionally, all people with IBD should have access to an individual dietitian with experience in IBD and nutritional evaluation as part of a multidisciplinary approach (10, 12).

Nutritional assessment includes a combination of history-taking, clinical evaluation that includes anthropometry, laboratory tests, and assessment of the energy balance. This can be calculated based on the caloric intake (estimated by collecting food intake records) and energy expenditure of patients with IBD. Although the best estimation of energy expenditure can be measured by indirect calorimetry or predictive formulas, in current clinical practice, the Harris–Benedict formula (Basal Energy Expenditure [BEE]) in combination with the possible factors of stress (Total Energy Expenditure [TEE] Kcal/day = BEE × stress factors) could be considered (19). Consistent with the GLIM consensus criteria, our patient was diagnosed with malnutrition (15).

Sarcopenia is a syndrome characterized by progressive and generalized loss of skeletal muscle mass and strength. Its prevalence can range from 17 to 70% in patients with IBD and has a negative impact on surgical outcomes, hospitalization times, morbidity, and mortality (11, 20, 21). The gold-standard methods for assessing muscle mass are DEXA, CT, and MRI, but they have limited use in clinical practice due to high costs. Body Impedance Analysis (BIA) may be a valid alternative to DEXA due to lower cost. Muscle strength can be assessed with an HST using a standard dynamometer (22). Our patient had an altered grip strength test, but we did not perform BIA because it was not available at that time, nor did we perform CT or MRI evaluation of muscle mass due to their limited use in clinical practice. The absence of BIA, DXA, or CT/MRI is therefore a significant limitation of our nutritional assessment; for this reason, and in line with EWGSOP2 criteria, we classify this patient’s condition as probable sarcopenia. Handgrip strength and calf circumference were not formally reassessed at hospital discharge or at the 6-month follow-up visit, which limits our ability to quantify the evolution of muscle strength over the course of recovery.

In patients with IBD in whom nutritional deprivation has extended over a long time, rapid interventions to prevent refeeding syndrome are mandatory, particularly with respect to phosphate and thiamine intake (10, 12, 23). When thiamine deficiency is suspected, thiamine must be infused before or at the same time as intravenous glucose administration to prevent Wernicke’s encephalopathy.

Zinc deficiency in patients with IBD can occur in up to 50% of patients and is linked to worse clinical outcomes and complications (24). Accordingly, the ECCO recommendations suggest monitoring zinc at diagnosis and annually in patients with CD (12). In our hospitalized patients, zinc was supplemented (oral zinc bisglycinate 50 mg/day for a month) despite normal levels that may have been confounded by the inflammatory state.

Iron-deficiency anemia is the most frequent manifestation in active IBD (25); diagnostic criteria for iron deficiency depend on the level of inflammation. In the presence of active disease and inflammation (CRP > 10 mg/L), as in our patient, a serum ferritin level of up to 100 ug/L may still be consistent with iron deficiency (12, 26). Intravenous iron administration is more effective and shows a faster response than oral supplementation and should be considered first-line treatment in patients with clinically active IBD and hemoglobin levels below 100 g/L (10, 12, 27). Given the importance of elevated CRP levels associated with severe anemia and low iron levels, our patient firstly received intravenous iron, followed by oral iron supplementation.

Serum folate (vitamin B9) levels are significantly lower in patients with IBD than in controls (28, 29). Folate deficiency affects 20–60% of patients with IBD and can worsen anemia. Moreover, it could lead to hyperhomocysteinemia, which may help explain the increased incidence of thromboembolic events observed in patients with CD and UCs (30). The risk of megaloblastic anemia significantly increases below the serum folate cut-off of 7 nmoL/L (10), which was the level found in our patient. For this reason, oral supplementation was introduced.

Vitamin B12 deficiency is more common in patients with prolonged active ileal CD, ileal resections >20 cm, or those following a vegan diet compared with those with colonic CD or UC (31–34). Oral, sublingual, or intramuscular vitamin B12 supplementation improves status, although patients with ileal resections are generally less responsive to oral supplementation (12, 35). Due to the low serum vitamin B12 levels and hyporexia, our patient received an initial intramuscular injection and then sublingual supplementation.

Magnesium is involved in the immune response and gut health, and it contributes to maintaining a normal circadian rhythm and improving sleep quality (36). International consensus recommends measuring serum magnesium levels only when deficiency is suspected, primarily in patients with diarrhea (12), as in our patient.

Low serum vitamin D levels can be associated with more severe disease activity, a worse response to conventional and biologic therapies, a higher risk of IBD-related complications, and increased rates of comorbidities such as osteoporosis and infections (37, 38). International recommendations suggest that calcium/vitamin D supplementation should be prescribed both for the prevention of low bone mineral density (12) and for reducing inflammation, thereby lowering the risk of disease relapse (38, 39). Given the severe inflammatory activity, low serum vitamin D levels with normal serum calcium levels, and corticosteroid treatment, our patient was started on oral vitamin D supplementation alone.

In general, energy delivery in patients with IBD is similar to that of the healthy population; in cases of clinical suspicion of an increased energy requirement due to acute inflammation, an accurate determination of the increased energy requirement has to be made, while also evaluating the patient’s physical activity levels (10). In adult active IBD, protein requirements increase to 1.2–1.5 g/kg/day due to the proteolytic and catabolic response to active inflammation (10). Moreover, corticosteroids increase the net protein loss in adults (10). For both reasons, our patient underwent nutritional and dietitian assessments, deciding upon an increased protein intake.

Dietary interventions in patients with active IBD are considered complementary to medical therapy. In CD, ONS is the first step when medical nutrition is indicated, in addition to normal food, to address specific deficiencies (10–12). Moreover, in active IBD, when oral feeding is insufficient, enteral nutrition with formulas or liquids should be recommended and preferred over parenteral nutrition, unless completely contraindicated or when all nutritional needs cannot be met by the gut (10, 12).

Exclusive enteral nutrition (EEN) for 6–8 weeks reduces intestinal inflammation and promotes mucosal healing, which is effective for the induction of clinical and endoscopic remission, both in children and adults with mild-to-moderate CD (10–12, 40).

Parenteral nutrition (PN) could not be advantageous in IBD compared to other nutritional therapies (12, 41). The ESPEN guidelines recommend PN in patients with IBD who are temporarily unable to receive significant oral or enteral nutrients, or when bowel obstruction or other complications occur (10, 41). A meta-analysis of PN in the treatment of IBD revealed improvements in both disease activity (as measured by the Crohn’s disease activity index) and albumin levels, but no increase in body weight (41). Moreover, given the higher risk with PN (catheter-related infections, higher liver dysfunction rates) and the efficacy equivalence to EEN, European guidelines do not recommend PN as induction therapy for IBD (12, 42).

In this specific case, three distinct components of the clinical course should not be conflated. The nutritional outcome reflects the combined effect of minimal enteral feeding, oral nutritional supplements, and supplemental parenteral nutrition. The medical outcome reflects the response to corticosteroid induction and vedolizumab maintenance therapy. The surgical/perianal outcome reflects timely abscess drainage, seton placement, and subsequent seton removal. While interdependent, these outcomes are attributable to distinct interventions, and the favorable overall course of this patient should not be interpreted as evidence of nutritional therapy alone. The educational value of this case lies specifically in illustrating how malnutrition and probable sarcopenia can be recognized and addressed at the time of IBD diagnosis, in the practical coordination of a multidisciplinary team.

**Figure 4:**
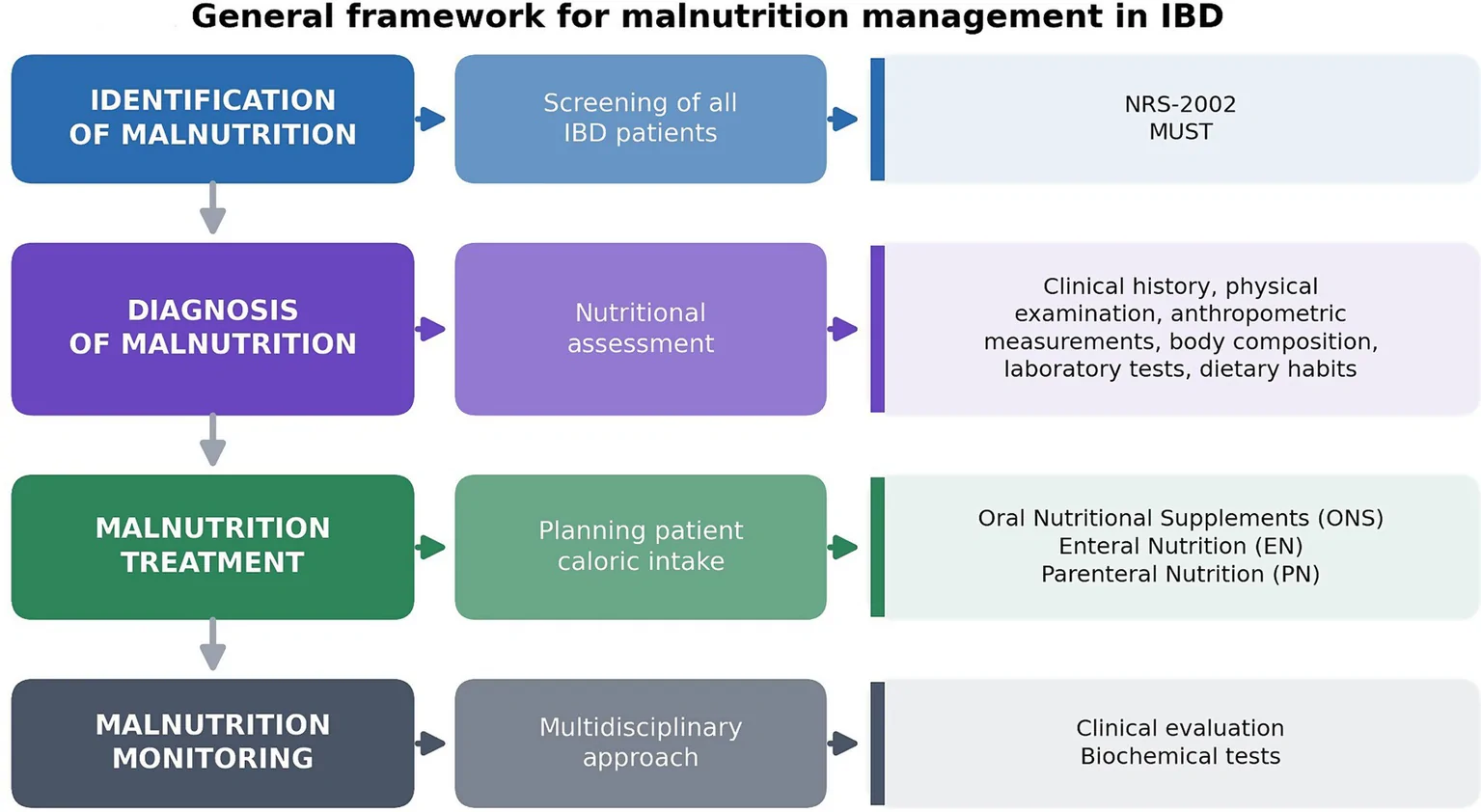
General framework for malnutrition management in IBD.

In conclusion, all patients with IBD should have access to an individual dietitian experienced in IBD and nutritional evaluation as part of a multidisciplinary approach to identify those at risk of malnutrition before its onset. Malnutrition and micronutrient deficiencies should be treated early upon identification to prevent complications, nutrition-related disorders and refeeding syndrome. Additionally, enteral nutrition should be preferred over parenteral nutrition in patients with active IBD and malnutrition, whenever possible. Moreover, ONS has to be considered an essential part of medical disease management, which can improve clinical outcomes. Both accurate periodic evaluation of nutritional status and nutritional therapy in all patients must be considered a keystone of IBD management (Figure 4).

## 4 Clinical follow-up

Six months after discharge, the patient underwent a clinical nutritional evaluation, indicating significant nutritional improvement with a further weight gain of 10 kg (BMI, 19.8 kg/m^2^) and a return to her previous body weight. PN was interrupted while continuing on a regular diet without ONS. Three months after hospital discharge, both a radiologic check with pelvic MRI and proctologic evaluation confirmed the perianal fistula healing so that the elastic seton could be removed, and the patient continued with biologic therapy.

In the following months, further blood tests showed persistent normalization of hemoglobin, iron, albumin, prealbumin, vitamin, and trace element levels, together with persistently normal CRP and low fecal calprotectin levels (120–180 ug/g). A colonoscopy performed 8 months after the initiation of biologic therapy showed a significant improvement in the prior endoscopic features (SES-CD 6). Hence, the Vedolizumab infusion every 8 weeks was continued. Handgrip strength was not re-evaluated at the 6-month follow-up visit, which focused on weight, BMI and biochemical nutritional markers. This is acknowledged as a limitation of the follow-up nutritional assessment.

## Data Availability

The raw data supporting the conclusions of this article will be made available by the authors, without undue reservation.

## References

[1] Nattagh-Eshtivani E Barghchi H Mansouri AH Rahbarinejd P Mokari-Yamchi A Barati M . The role of dietary factors in the prevention and management of inflammatory bowel disease: a comprehensive review. Nutr Metab (Lond). (2025) 22:157. doi: 10.1186/s12986-025-01068-y, 41423598 PMC12751218

[2] Hracs L Windsor JW Gorospe J Cummings M Coward S Buie MJ . Global evolution of inflammatory bowel disease across epidemiologic stages. Nature. (2025) 642:458–66. doi: 10.1038/s41586-025-08940-0, 40307548 PMC12158780

[3] Peyrin-Biroulet L Loftus EV Colombel JF Sandborn WJ. The natural history of adult Crohn’s disease in population-based cohorts. Am J Gastroenterol. (2010) 105:289–97. doi: 10.1038/ajg.2009.579, 19861953

[4] Michels KB Schulze MB. Can dietary patterns help us detect diet-disease associations? Nutr Res Rev. (2005) 18:241–8. doi: 10.1079/NRR2005107, 19079908

[5] Liu J Ge X Ouyang C Wang D Zhang X Liang J . Prevalence of malnutrition, its risk factors, and the use of nutrition support in patients with inflammatory bowel disease. Inflamm Bowel Dis. (2022) 28:S59–66. doi: 10.1093/ibd/izab345, 34984471 PMC10686604

[6] Bezzio C Brinch D Ribaldone DG Cappello M Ruzzon N Vernero M . Prevalence, risk factors and association with clinical outcomes of malnutrition and sarcopenia in inflammatory bowel disease: a prospective study. Nutrients. (2024) 16:3983. doi: 10.3390/nu16233983, 39683376 PMC11643262

[7] Barberio B Bertin L Facchin S Bonazzi E Cusano S Romanelli G . Dietary interventions and Oral nutritional supplementation in inflammatory bowel disease: current evidence and future directions. Nutrients. (2025) 17:1879. doi: 10.3390/nu17111879, 40507145 PMC12157630

[8] Reznikov EA Suskind DL. Current nutritional therapies in inflammatory bowel disease: improving clinical remission rates and sustainability of long-term dietary therapies. Nutrients. (2023) 15:668. doi: 10.3390/nu15030668, 36771373 PMC9920576

[9] Spooren CEGM Wintjens DSJ de Jong MJ van der Meulen-de Jong AE Romberg-Camps MJ Becx MC . Risk of impaired nutritional status and flare occurrence in IBD outpatients. Dig Liver Dis. (2019) 51:1265–9. doi: 10.1016/j.dld.2019.05.024, 31213405

[10] Bischoff SC Bager P Escher J Forbes A Hébuterne X Hvas CL . ESPEN guideline on clinical nutrition in inflammatory bowel disease. Clin Nutr. (2023) 42:352–79. doi: 10.1016/j.clnu.2022.12.004, 36739756

[11] Potcovaru CG Filip PV Neagu OM Diaconu LS Salmen T Cinteza S . Diagnostic criteria and prognostic relevance of sarcopenia in patients with inflammatory bowel disease—a systematic review. J Clin Med. (2023). 10.3390/jcm12144713 PMC10381373 37510827

[12] Svolos V Gordon H Lomer MCE Aloi M Bancil A Day AS . European Crohn’s and colitis organisation consensus on dietary management of inflammatory bowel disease. J Crohns Colitis. (2025) 19:jjaf122. doi: 10.1093/ecco-jcc/jjaf122, 40650933

[13] Nutrition Risk Screening. (2002). Predicts risk of malnutrition in hospitalized patients, recommended by ACG guidelines. (NRS-2002). Available online at: https://www.mdcalc.com/calc/4012/nutrition-risk-screening-2002-nrs-2002.

[14] Malnutrition Universal Screening Tool (MUST). Identifies patients who are malnourished or at risk of malnutrition. MDCalc (2003). Available online at: https://www.mdcalc.com/calc/10190/malnutrition-universal-screening-tool-must.

[15] Cederholm T Jensen GL Correia MITD Gonzalez MC Fukushima R Higashiguchi T . GLIM criteria for the diagnosis of malnutrition – a consensus report from the global clinical nutrition community. Clin Nutr. (2019) 38:1–9. doi: 10.1016/j.clnu.2018.08.002, 30181091

[16] Vadan R Gheorghe LS Constantinescu A Gheorghe C. The prevalence of malnutrition and the evolution of nutritional status in patients with moderate to severe forms of Crohn’s disease treated with infliximab. Clin Nutr. (2011) 30:86–91. doi: 10.1016/j.clnu.2010.07.019, 20719413

[17] Casanova MJ Chaparro M Molina B Merino O Batanero R Dueñas-Sadornil C . Prevalence of malnutrition and nutritional characteristics of patients with inflammatory bowel disease. J Crohns Colitis. (2017) 11:1430–9. doi: 10.1093/ecco-jcc/jjx102, 28981652

[18] Viganò C Palermo A Mulinacci G Pirola L Losco A Meucci G . Prevalence of disease-related malnutrition and micronutrients deficit in patients with inflammatory bowel disease: a multicentric cross-sectional study by the GSMII (inflammatory bowel disease study group). Inflamm Bowel Dis. (2024) 30:1112–20. doi: 10.1093/ibd/izad146, 37536282

[19] Miles JM. Energy expenditure in hospitalized patients: implications for nutritional support. Mayo Clin Proc. (2006) 81:809–16. doi: 10.4065/81.6.809, 16770981

[20] Yuan S Larsson SC. Epidemiology of sarcopenia: prevalence, risk factors, and consequences. Metabolism. (2023) 144:155533. doi: 10.1016/j.metabol.2023.155533, 36907247

[21] Liu S Ding X Maggiore G Pietrobattista A Satapathy SK Tian Z . Sarcopenia is associated with poor clinical outcomes in patients with inflammatory bowel disease: a prospective cohort study. Ann Transl Med. (2022) 10:367. doi: 10.21037/atm-22-1126, 35433981 PMC9011317

[22] Cruz-Jentoft AJ Bahat G Bauer JM Boirie Y Bruyère O Cederholm T . Sarcopenia: revised European consensus on definition and diagnosis. Age Ageing. (2019) 48:16–31. doi: 10.1093/ageing/afy169, 30312372 PMC6322506

[23] McMillan DC Maguire D Talwar D. Relationship between nutritional status and the systemic inflammatory response: micronutrients. Proc Nutr Soc. (2019) 78:56–67. doi: 10.1017/S0029665118002501, 30220267

[24] Almuhaya H Alsalamah R Sallam A Alonazy A AlAwwad A Mohamed G . Prevalence and impact of zinc deficiency on clinical outcomes in inflammatory bowel disease. Nutrients. (2025) 17:3378. doi: 10.3390/nu17213378, 41228451 PMC12610508

[25] Akhuemonkhan E Parian A Miller K Hanauer S Hutfless S. Prevalence and screening for anemia in mild to moderate Crohn’s disease and ulcerative colitis in the United States, 2010–2014. BMJ Open Gastroenterol. (2017) 4:e000155. doi: 10.1136/bmjgast-2017-000155, 28944071 PMC5609082

[26] Niepel D Klag T Malek NP Wehkamp J. Practical guidance for the management of iron deficiency in patients with inflammatory bowel disease. Ther Adv Gastroenterol. (2018) 11:1756284818769074. doi: 10.1177/1756284818769074, 29760784 PMC5946590

[27] Bergamaschi G Castiglione F D’Incà R Astegiano M Fries W Milla M . Prevalence, pathogenesis and management of anemia in inflammatory bowel disease: an IG-IBD multicenter, prospective, and observational study. Inflamm Bowel Dis. (2023) 29:76–84. doi: 10.1093/ibd/izac054, 35366312

[28] Valvano M Capannolo A Cesaro N Stefanelli G Fabiani S Frassino S . Nutrition, nutritional status, micronutrients deficiency, and disease course of inflammatory bowel disease. Nutrients. (2023) 15:3824. doi: 10.3390/nu15173824, 37686856 PMC10489664

[29] Pan Y Liu Y Guo H Jabir MS Liu X Cui W . Associations between folate and vitamin B12 levels and inflammatory bowel disease: a meta-analysis. Nutrients. (2017) 9:382. doi: 10.3390/nu9040382, 28406440 PMC5409721

[30] Yakut M Ustün Y Kabaçam G Soykan I. Serum vitamin B12 and folate status in patients with inflammatory bowel diseases. Eur J Intern Med. (2010) 21:320–3. doi: 10.1016/j.ejim.2010.05.007, 20603044

[31] Gomollòn F Gargallo CJ Muñoz JF Vicente R Lue A Mir A . Oral cyanocobalamin is effective in the treatment of vitamin B12 deficiency in Crohn’s disease. Nutrients. (2017) 9:308. doi: 10.3390/nu9030308, 28335526 PMC5372971

[32] Ward MG Kariyawasam VC Mogan SB Patel KV Pantelidou M Sobczyńska-Malefora A . Prevalence and risk factors for functional vitamin B12 deficiency in patients with Crohn’s disease. Inflamm Bowel Dis. (2015) 21:2839–47. doi: 10.1097/MIB.0000000000000559, 26296064

[33] Bakaloudi DR Halloran A Rippin HL Oikonomidou AC Dardavesis TI Williams J . Intake and adequacy of the vegan diet. A systematic review of the evidence. Clin Nutr. (2021) 40:3503–21. doi: 10.1016/j.clnu.2020.11.035, 33341313

[34] Madanchi M Fagagnini S Fournier N Biedermann L Zeitz J Battegay E . The relevance of vitamin and iron deficiency in patients with inflammatory bowel diseases in patients of the Swiss IBD cohort. Inflamm Bowel Dis. (2018) 24:1768–79. doi: 10.1093/ibd/izy054, 29669023

[35] Battat R Kopylov U Szilagyi A Saxena A Rosenblatt DS Warner M . Vitamin B12 deficiency in inflammatory bowel disease: prevalence, risk factors, evaluation, and management. Inflamm Bowel Dis. (2014) 20:1120–8. doi: 10.1097/MIB.0000000000000024, 24739632

[36] Gilca-Blanariu GE Trifan A Ciocoiu M Popa IV Burlacu A Balan GG . Magnesium—a potential key player in inflammatory bowel disease? Nutrients. (2022) 14:1914. doi: 10.3390/nu14091914 PMC9102374 35565881

[37] Garg Y Sharma N Narang RK Singh A. The vitamin D and IBD nexus: immune links and therapeutic prospects. J Am Nutr Assoc. (2026) 45:1–5. doi: 10.1080/27697061.2025.2518117, 40551677

[38] Dell’Anna G Fanizzi F Zilli A Furfaro F Solitano V Parigi TL . The role of vitamin D in inflammatory bowel diseases: from deficiency to targeted therapeutics and precise nutrition strategies. Nutrients. (2025) 17:2167. doi: 10.3390/nu17132167, 40647273 PMC12252289

[39] Goh J O’Morain CA. Review article: nutrition and adult inflammatory bowel disease. Aliment Pharmacol Ther. (2003) 17:307–20. doi: 10.1046/j.1365-2036.2003.01482.x, 12562443

[40] Comeche JM Caballero P Gutierrez-Hervas A García-Sanjuan S Comino I Altavilla C . Enteral nutrition in patients with inflammatory bowel disease. Systematic review, meta-analysis, and meta-regression. Nutrients. (2019) 11:2657. doi: 10.3390/nu11112657, 31689999 PMC6893586

[41] Comeche JM Comino I Altavilla C Tuells J Gutierrez-Hervas A Caballero P. Parenteral nutrition in patients with inflammatory bowel disease systematic review, meta-analysis and meta-regression. Nutrients. (2019) 11:2865. doi: 10.3390/nu11122865, 31766687 PMC6950216

[42] Comerlato PH Stefani J Viana LV. Mortality and overall and specific infection complication rates in patients who receive parenteral nutrition: systematic review and meta-analysis with trial sequential analysis. Am J Clin Nutr. (2021) 114:1535–45. doi: 10.1093/ajcn/nqab218, 34258612

